# Devilish Details in Apes and Atoms

**DOI:** 10.1100/tsw.2001.6

**Published:** 2001-02-09

## Abstract

Nature leads with a preview of an upcoming workshop on sequencing the ape genome. Science leads off this week with a story about a new measurement that comes tantalizingly close to overturning the longstanding Standard Model of particle physics.

The Genes and Minds Initiative (GEMINI), is a workshop scheduled for next month in Tokyo to discuss sequencing the ape genome. *Nature* reports that organizers hope the meeting will launch an international effort to compare the genomes of apes and humans, which are approximately 99% equivalent. Engaging in the usual genomic hyperbole, scientists quoted in *Nature* predicted that such a comparison might establish why apes are resistant to Alzheimer's Disease and AIDS. But before they cure AIDS, the researchers have to come up with some cash. So far, the two Japanese groups leading the effort - the National Institute of Genetics (NIG) and the Institute of Physical and Chemical Research (RIKEN) - have been forced to fund the project by skimming money from other programs, reports *Nature*. Although the project is expected to be cheap by modern standards, a mere $100 million, say *Nature*'s sources, recent history suggests that the altruistic sequencing project will have trouble locating a ready supply of cash. A RIKEN project to compare chimpanzee and human genomic sequences announced last year has not yet found funding, reports *Nature*. *Science* did not cover this story.

*Science* leads with hints that the Standard Model of particle physics, and, by extension, the standard model of pretty much everything, might be...gasp...wrong. As *Science* went to press, physicists at Brookhaven National Laboratory in New York are preparing to announce the results of a three-year study of smashed protons. According to the physicists, a rare cousin of the electron called a muon that is created in the atomic collisions is not behaving in a predictable way. Physicists can measure the muon's small magnetic moment by directing the particles into the magnetic field of a superconducting magnet, which spins the muon by an amount proportional to the particle's magnetic moment. The muons then decay into other particles and the scientists deduce how much the particle twisted by observing the decay products, says *Science*. The results show that the theoretical value and the experimental value disagree by about four parts per million, reports *Science*. Close but no cigar. Although there is a distinct chance that undetected errors could change the result, reports *Science*, physicists at Brookhaven are already suggesting that the Standard Model's days are numbered. *Nature* did not cover this story.

In other news, *Nature* reported on the "bombastic" report that calls for doubling the U.S. expenditures for science and technology "in the name of national security." Although the report, prepared by a commission led by former senators Warren Rudman (Republican-New Hampshire) and Gary Hart (Democrat-Colorado), claimed that U.S. science was "in crisis," it offered little evidence to support the claim, reports *Nature*.
